# Accuracy and Outcomes of Sentinel Lymph Node Biopsy in Male with Breast Cancer: A Narrative Review and Expert Opinion

**DOI:** 10.3390/curroncol31120557

**Published:** 2024-11-27

**Authors:** Calogero Cipolla, Vittorio Gebbia, Eleonora D’Agati, Martina Greco, Chiara Mesi, Giuseppa Scandurra, Daniela Sambataro, Maria Rosaria Valerio

**Affiliations:** 1 Department of Biomedicina, Neuroscienze e Diagnostica Avanzata, University of Palermo, 90127 Palermo, Italy; calogero.cipolla@unipa.it (C.C.); dagatieleonora@gmail.com (E.D.); doc.martinagreco@gmail.com (M.G.); chiaramesi95@gmail.com (C.M.); mariarosaria.valerio@unipa.it (M.R.V.); 2 Breast Unit, AOUP Policlinico Paolo Giaccone, 90127 Palermo, Italy; 3 Department of Medicine and Surgery, Kore University of Enna, 94019 Enna, Italy; giuseppa.scandurra@unikore.it (G.S.); daniela.sambataro@unikore.it (D.S.); 4 Medical Oncology Unit, Cdc Torina, 90100 Palermo, Italy; 5 Medical Oncology Unit, AOUP Policlinico Paolo Giaccone, 90127 Palermo, Italy; 6 Medical Oncology Unit, Ospedale Cannizzaro, 94126 Catania, Italy; 7 Ospedale Umberto I Enna, 94019 Enna, Italy

**Keywords:** male breast cancer, sentinel lymph node biopsy, survival

## Abstract

Male breast cancer (MBC) is a rare disease, accounting for less than 1% of all breast cancer cases. Sentinel lymph node biopsy (SLNB) has emerged as a less invasive alternative to axillary lymph node dissection (ALND) for axillary staging in breast cancer, offering reduced morbidity and comparable accuracy. However, the application of SLNB in MBC remains underexplored, with limited male-specific data and treatment protocols often extrapolated from female breast cancer studies. Available evidence suggests that SLNB in men demonstrates high diagnostic accuracy, with low false-negative rates and a high sentinel lymph node identification rate. Despite this, there is ongoing debate about its long-term impact on clinical outcomes, particularly for patients with sentinel node metastasis, where ALND may still provide superior survival outcomes in some cases. Predictive tools are being developed to identify better patients who may benefit from SLNB alone, potentially reducing the need for more invasive procedures. As the role of SLNB continues to evolve in MBC management, further prospective research is needed to refine its application and assess its long-term oncologic outcomes.

## 1. Introduction

Male breast cancer (MBC) is a rare malignancy, accounting for less than 1% of all breast cancer (BC) cases and cancer-related deaths in men [1,2,3]. While the lifetime risk of developing BC is significantly lower in men—approximately 1 in 1000 compared to 1 in 8 for women—this rarity has led to a lack of focused research and clinical trials for MBC. As a result, treatment protocols for men are often extrapolated from studies conducted on female BC [4]. Recent data suggest that MBC incidence may be increasing, potentially due to improved detection and reporting [5].

Axillary staging is crucial for determining the prognosis and guiding treatment of BC, regardless of gender. Historically, axillary lymph node dissection (ALND) has been the gold standard for staging, but it is associated with considerable morbidity, including lymphedema, pain, and restricted shoulder motion [6].

In women, sentinel lymph node biopsy (SLNB) has become a less invasive alternative to axillary lymph node dissection (ALND) [7,8,9]. By targeting the first lymph node(s) to which cancer cells are most likely to spread—the sentinel node—SLNB can accurately predict the overall involvement of axillary lymph nodes. This technique helps avoid unnecessary ALND in patients with clinically negative axillary nodes, thereby reducing surgery-related complications. This approach has been widely adopted in managing female BC, demonstrating high success and low false-negative rates [7,8]. However, SLNB may be omitted in some patient subpopulations, such as older ones and those with early-stage hormone-positive tumors [10]. In such cases, a multidisciplinary evaluation concerning radiotherapy is mandatory.

Although consensus exists on some general aspects of protocols for identifying sentinel lymph nodes in BC, an agreement has yet to be reached on all technical and practical details [11]. Generally, the procedure involves using an interstitial tracer injection, such as ^99m^Tc-albumin nano colloid, for preoperative scintigraphy imaging and intraoperative gamma probe localization for surgical removal of the radioactive lymph nodes that are detected. Using lymphoscintigraphy plus a vital dye achieves a very high detection rate (up to 98%). The optimal procedure should include the removal of three nodes.

Despite its widespread use among women, the role of SLNB in male breast cancer (MBC) is unclear because few large-scale studies focus on men. Specifically, its accuracy and oncological outcomes have been minimally researched [12,13]. However, due to the similarities in tumor biology between male and female BC, SLNB is considered equally important for staging and managing MBC [13,14].

This narrative review evaluates the evidence supporting sentinel lymph node biopsy (SLNB) in men with BC. It focuses on its accuracy, impact on surgical and oncological outcomes, and potential directions for future research.

## 2. Diagnostic Accuracy of SNB in Men

A recent landmark meta-analysis by Parpex et al. provides critical insights into the diagnostic performance of SLNB in MBC [5]. This study synthesized data from 12 retrospective studies, evaluating the SLNB identification rate and the false-negative rate (FNR) in 153 patients for SLNB identification and 50 patients for FNR assessment. The results were promising, with a pooled SLNB identification rate of 99.0% and a narrow confidence interval (97.1–101%), indicating that the procedure reliably locates SLN in the vast majority of cases. Furthermore, across the five studies reporting this outcome, the FNR for SLNB was remarkably low, with some studies even reporting a rate of 0%. This suggests that when SLNB is performed in men, it is highly effective in detecting axillary metastases, minimizing the risk of missing nodal involvement [5].

While the existing data point to the strong diagnostic accuracy of SLNB in men, the relatively small sample sizes and retrospective design of the studies included in this meta-analysis highlight the need for larger prospective trials. These future studies will be crucial in further establishing the role of SLNB in the clinical management of MBC and ensuring that the findings can be generalized to broader patient populations.

## 3. Impact on Surgical and Oncologic Outcomes

Some studies have explored the comparative effectiveness of SLNB and ALND in MBC, with mixed results. These studies highlight the benefits of SLNB in reducing surgical morbidity but also indicate that ALND may provide superior outcomes in specific cases (Table 1) [14,15,16,17].

Shang et al. reported on 92 patients with early-stage disease and clinically negative nodes [16]. They analyzed outcomes across three groups: SLNB alone, ALND alone, and SLNB followed by ALND. Overall, 95% and 91% of patients had estrogen- and progestin-positive breast cancer, respectively, with only 11% showing HER2 overexpression. After a median follow-up of 3.5 years, the study found no significant differences in 5-year survival rates between the SLNB and ALND groups, with fewer complications in the SLNB group. A total of 157 patients underwent ALND, but only half had positive nodes, leading to unnecessary complications such as lymphedema, numbness, and dyskinesia. This was likely due to the study’s retrospective nature, which included patients treated during the transition from ALND to sentinel node biopsy. These findings suggest that SLNB is a safe and effective alternative to ALND for men with clinically negative axillary nodes, helping to reduce surgical morbidity.

Data from Chung et al. indicated that men with T1–T2 BC and 1–2 positive sentinel nodes had significantly better 5-year overall survival (OS) when treated with axillary lymph node dissection (ALND) compared to sentinel lymph node biopsy (SLNB) alone [15]. In this study, patients with positive sentinel nodes who underwent SLNB alone faced a higher risk of poorer survival outcomes. This raises concerns about applying SLNB-only strategies from female BC studies to male patients. The study suggests that ALND may still be necessary for MBC patients with sentinel node metastasis to ensure optimal long-term survival.

Leone et al. conducted a population-based study focusing on locoregional treatment and the number of lymph nodes examined (LNE) [14]. Estrogen-positive patients made up 82.9%, and progestin-positive ones were 74.4%. The study found no significant difference in OS between patients with 1–5 LNE, representing SLNB, and those with more than 5 LNE, typical of ALND (Table 1). This finding suggests that for node-negative patients, SLNB might suffice for axillary staging, potentially reducing unnecessary morbidity without compromising survival outcomes. However, patients who did not undergo breast surgery or had fewer lymph nodes examined had worse prognoses, highlighting the need for careful patient selection.

Carter et al. found a significant increase in sentinel lymph node biopsy (SLNB) use from 2006 to 2016 [17]. Conversely, axillary lymph node dissection (ALND) usage declined during the same period. Nearly 96% of patients had an estrogen-positive BC, and 83.4% had a progestin-positive tumor. Among patients who underwent SLNB, survival analysis revealed that those diagnosed in the later period (2012–2016) had worse survival compared to those diagnosed earlier, with a hazard ratio of 1.46 for worse survival (*p* < 0.01). The median survival for patients treated between 2006 and 2011 was 130.5 months, while the median survival for those treated after 2011 could not be estimated due to insufficient follow-up. The study suggested that the poorer outcomes in the later cohort may be partially explained by the more advanced clinical (cT2: 68.5% vs. 55.8%, *p* < 0.01) and pathologic T stages (pT2: 58.1% vs. 50.4%, *p* < 0.01), as well as lower rates of adjuvant chemotherapy (27.2% vs. 35.1%, *p* < 0.01) [14]. Further insights from Chen et al. offer valuable data on predicting axillary lymph node metastasis (ALNM) in men, which can help guide using SLNB [18]. Using a cohort of 2,610 men from the Surveillance, Epidemiology, and End Results (SEER) database, Chen et al. developed a nomogram to predict the likelihood of ALNM. The study found that factors such as age, tumor location, tumor stage, pathological type, and histologic grade were significantly associated with ALNM. The nomogram demonstrated strong predictive performance with an area under the curve (AUC) of 0.846 (95% CI: 0.825–0.867) in the training cohort and 0.848 (95% CI: 0.819–0.877) in the validation cohort. These results suggest that the nomogram could be a valuable clinical tool for identifying patients at low risk of ALNM, potentially allowing some men to avoid SLNB without compromising overall survival.

Taken together, these studies provide a nuanced understanding of SLNB’s role in MBC management. While SLNB offers a less invasive option with fewer complications, ALND may still be required for optimal oncologic outcomes, especially in patients with sentinel node metastasis. More prospective studies are needed to clarify the role of SLNB in MBC, particularly for those with advanced disease.

## 4. Expert Opinion and Future Directions

MBC is a rare and under-researched disease. It is often diagnosed later than its female counterpart, contributing to poorer survival outcomes [2,3]. In recent years, SLNB has emerged as a critical tool for axillary staging in MBC. It has very high diagnostic accuracy and may reduce surgical morbidity compared to ALND [5].

Despite these promising results, the outcomes of SLNB in men have been underexplored in clinical research. A recent meta-analysis by Parpex et al. addressed the accuracy of SLNB but left essential questions unanswered regarding long-term oncologic outcomes for MBC. Our review highlights areas needing further study, including the clinical impact of SLNB and specific challenges in applying it to male subpopulations. This ongoing gap underscores the need to integrate male-focused insights and clinical data to ensure the benefits of SLNB effectively translate into MBC management. These challenges are compounded by differences in tumor presentation, biological behavior, and metastatic patterns in men, which may influence the effectiveness and reliability of SLNB as a predictor of axillary involvement. Currently, MBC patients face inconsistent treatment approaches, often receiving ALND despite the high diagnostic accuracy of SLNB, indicating a need for better patient selection criteria specific to male physiology.

Additionally, since MBC is often diagnosed at later stages, questions arise about whether SLNB is as suitable for men with locally advanced disease as it is for early-stage cases of female BC [19]. This ongoing gap highlights the need to integrate male-focused insights and clinical data to ensure the benefits of SLNB are effectively applied in MBC management. As noted by Pratt et al., inconsistencies in the surgical management of MBC persist, with many cases still relying on ALND despite the proven benefits of SLNB [19]. This discrepancy presents a fundamental challenge: applying the clinical advantages of SLNB in female BC to its usage in men.

**Figure 1:**
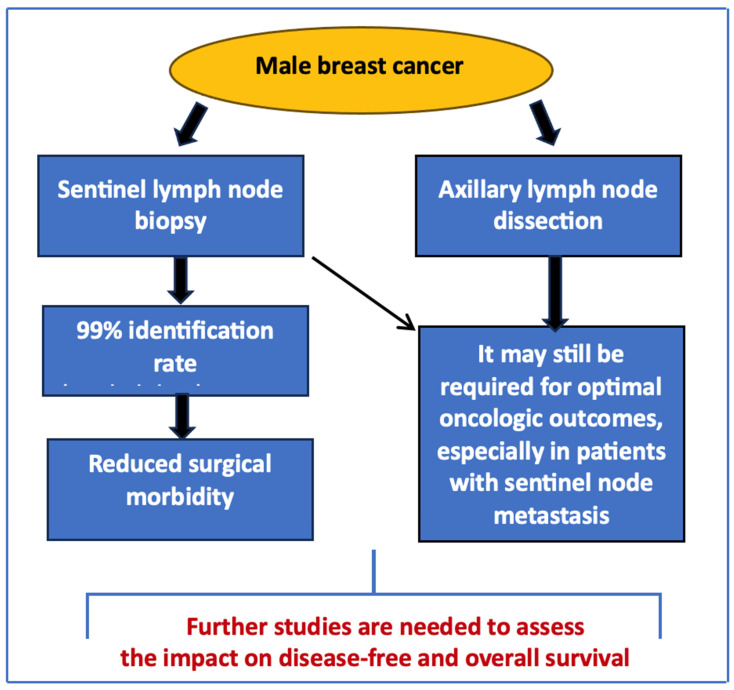
SLNB and ALND in MBC.

Indeed, the sporadic nature of MBC and its frequent misdiagnosis contribute to elevated mortality rates. This highlights the need for increased awareness among healthcare professionals, especially primary care physicians, who often see patients with MBC [20,21]. Early diagnosis is crucial since clinical presentations can differ, and there may be no apparent risk factors compared to female BC. In this scenario, SLNB provides a reliable and less invasive staging option, particularly for men with clinically node-negative disease (Figure 1).

Looking forward, several key areas require further exploration. Firstly, robust predictive models and nomograms tailored to men with MBC need to be developed to optimize SLNB’s clinical utility. These tools ideally incorporate factors specific to MBC, such as age, hormone receptor status, and tumor biology, to better stratify patients for SLNB versus ALND. Additionally, standardizing SLNB protocols to account for the variability in male axillary anatomy may improve procedure accuracy and reduce false-negative rates. As SLNB continues to gain acceptance in MBC, large-scale prospective trials are needed to solidify its role, particularly in terms of long-term survival outcomes and its applicability in advanced-stage disease. The association between SLNB and clinical outcomes remains an area of significant interest, given the inconclusive results reported to date. For example, it has been suggested that SLNB alone may not be sufficient in patients with sentinel node metastasis, while ALND is still associated with superior overall survival [15]. This raises important questions about patient selection and whether certain high-risk groups, such as those with larger tumors or more aggressive disease, might benefit more from a combined SLNB-ALND approach.

Further research topics may include the role of sentinel node biopsy in ductal carcinoma in situ (DCIS), the presence of sentinel node micro-metastasis, and the criteria for axillary lymph node dissection. This includes minimizing axillary surgery in men with one to two positive sentinel lymph nodes (SLNs) who are undergoing breast-conserving surgery (BCS) and radiation therapy, as well as in men with clinically node-positive breast cancer who respond well to neoadjuvant systemic therapy.

The low incidence of BC in men presents challenges for conducting prospective studies on these topics, emphasizing the importance of retrospective studies to validate the relevance of data from female patients. Future BC research should promote the inclusion of men in study designs.

Sentinel lymph node biopsy is generally not recommended before surgery for ductal carcinoma in situ (DCIS) unless there is a strong suspicion of microinvasion. Such suspicion can arise in cases of high-grade lesions larger than 2 cm, where mastectomy is performed due to large lesions like multiple foci of microcalcifications or when DCIS is associated with a large nodular component lesion [22].

The role of axillary surgery in sentinel lymph node micro-metastasis cases has been studied [22,23,24,25]. The prospective multicenter SENOMIC225 study enrolled 566 patients with BC and sentinel lymph node micro-metastases (undergoing breast-conserving surgery or mastectomy) not undergoing completion with ALDN [23]. The analysis of the primary endpoint EFS, at a median follow-up of 38 months, showed 3-year EFS rates of 96.2%. Stratifying patients based on the type of breast surgery, the 3-year EFS rate was 93.8% vs. 97.8% for those who underwent mastectomy vs. breast-conserving surgery, respectively. It is essential to underline that the clinicopathological characteristics of patients who underwent mastectomy were more unfavorable. The risk of events was higher in patients who underwent mastectomy without subsequent radiotherapy compared to patients who underwent breast-conserving surgery. In 2013, a multicenter, non-inferiority phase III study (IBCSG 23-01) was published in which 934 patients diagnosed with BC ≤ cT2 and cN0 and with micro-metastases in 1 or more sentinel lymph nodes were randomized to receive ALDN or not [24,25]. This trial demonstrated equivalent disease-free survival (76.8 vs. 74.9% in the treatment arm), a virtually comparable incidence of axillary recurrence (1% vs. 2%), and a 3-fold decrease in severe complications (lymphedema and motor neuropathy) in the observation group.

A second randomized trial on 247 patients (AATRM 048) achieved equivalent results at a median follow-up of 5 years [26]. In light of these data, ALDN is not indicated regardless of the type of breast surgery as observation was reported to be non-inferior to treatment, both in terms of DFS and regional recurrences, with a potential reduction in morbidity. In the case of sentinel lymph node micro-metastases, the benefit/harm balance appears to favor observation alone, as it was found to be non-inferior to ALDN in terms of recurrence-free survival rates or regional recurrence rates, compared with a significant reduction in severe complications such as lymphedema and motor neuropathy. In patients with cT1–2 and cN0 invasive BC with sentinel lymph node micro-metastases, the omission of axillary dissection should be considered regardless of the type of breast surgery.

The ACOSOG Z0011 randomized study and a meta-analysis have examined the de-escalation of axillary surgery in patients with 1–2 positive sentinel lymph nodes (SLNs) undergoing breast-conserving surgery (BCS) and radiation therapy [27,28]. The ACOSOG Z0011 study reported on 856 patients with cT1–2 BC who had one or two positive sentinel lymph nodes according to histology [24]. It is important to note that the study closed early due to difficulties recruiting patients (40% of the target), and 80% of those involved were considered low-risk (T1, hormone-responsive). The survival data were similar in both receptor-positive and receptor-negative groups. Additionally, many participants were postmenopausal; micro-metastases accounted for about half of the cases, and there needed to be more follow-up for many patients, with missing data on radiotherapy. The activation of six randomized studies underscores the necessity for conclusive confirmation of the ACOSOG Z0011 study.

A systematic review with meta-analysis of six retrospective studies and one randomized trial on 8864 patients did not find statistically significant differences in terms of OS and DFS at a median follow-up of 40 months, while a substantial decrease in the incidence of recurrence and lymphedema was recorded in patients treated only with LS [28].

Recently, a multicenter randomized non-inferiority trial, SINODAR ONE, involved 889 women with unilateral T1/2 BC who underwent surgery (either quadrantectomy or mastectomy) and had one or two macro metastatic sentinel lymph nodes [29]. The trial reported a five-year recurrence rate of 3.3% in the experimental group (BLS only) and 6.9% in the standard group (BLS + DA). Only one axillary recurrence was recorded in each treatment arm at a median follow-up of 34 months. No differences in OS were observed, with rates of 98.9% for the experimental group and 98.8% for the standard group (*p* = 0.9). In patients with BC cT1–2 and cN0, with macro-metastases in 1–2 sentinel lymph nodes, undergoing conservative surgery, and treated with breast radiotherapy and systemic therapy, the omission of ALDN can be considered.

Several studies have examined the de-escalation of axillary surgery in patients with clinically node-positive BC who respond well to neoadjuvant systemic therapy [30,31]. For patients with cN1 before neoadjuvant systemic therapy and subsequent clinical–radiological negativity after treatment, omitting axillary dissection may be considered if one or more sentinel lymph nodes, possibly identified using double tracers and/or clips, are found negative. On the other hand, the long-term follow-up results of a prospective-randomized trial suggest that regional node radiotherapy does not increase the risk of axillary failure in selected patients with early-stage invasive breast cancer (cT ≤ 3 cm, cN0) and pN1(sn) [32]. Axillary radiotherapy should be an alternative treatment for selected patients with sentinel lymph node metastases. Moreover, there is a growing need for the development of male-specific treatment guidelines, particularly as current therapeutic approaches, including surgical and adjuvant treatments, are derived mainly from female BC data. While hormonal therapy, particularly tamoxifen, remains a cornerstone of MBC treatment, the role of adjuvant chemotherapy, particularly in the context of SLNB and node-positive disease, is less well defined [33]. Given SLNB’s potential to accurately stage axillary involvement while sparing men from more invasive surgery, future guidelines should aim to provide specific criteria that address both clinical and quality-of-life outcomes for male patients. This reinforces the need for research focusing on MBC-specific outcomes regarding treatment efficacy and quality of life.

In conclusion, while SLNB has shown great promise in reducing morbidity and providing accurate axillary staging in men with breast cancer, its full potential is yet to be realized. Developing male-specific clinical trials and treatment guidelines is crucial to optimizing outcomes for this underrepresented population. As we build on the current evidence, the consistent and evidence-based use of SLNB, combined with an interdisciplinary approach to MBC treatment, will be pivotal in ensuring male patients receive the individualized care they deserve.

## Figures and Tables

**Table 1:** Comparative effectiveness of SNB and ALND in men with BC.

Study	Design	Population	Key Results	Key Findings
Shang et al. (2023) [16]	Multicenter retrospective study	92 men with early-stage MBC and clinically negative nodes	- 5-year DFS: 83.9% (SLNB) vs. 82.6% (ALND) - 5-year OS: 93.5% (SLNB) vs. 87% (ALND) - Complications: 1 mild lymphedema case (SLNB) vs. 8 complications (ALND)	SLNB showed non-inferiority to ALND for DFS and OS in men with clinically negative axillary nodes, with fewer complications in the SLNB group
Chung et al. (2023) [15]	Retrospective using the NCDB	1203 men with T1–T2 breast cancer and 1–2 positive sentinel nodes	- 5-year OS: 83.8% (ALND) vs. 76.0% (SLNB), *p* = 0.0104	ALND was associated with superior survival compared to SLNB alone in men with sentinel node metastasis, suggesting SLNB alone may not be sufficient
Leone et al. (2017) [14]	Population-based using the SEER database	1263 men with early-stage (T1abcN0M0) breast cancer	- 10-year OS: 85.1% (SLNB) vs. 66.5% (ALND)	SLNB may be adequate for node-negative patients, reducing unnecessary morbidity without compromising survival outcomes
Carter et al. (2021) [17]	Retrospective using NCDB	2646 men with clinically node-negative (cN0) breast cancer	- SLNB use increased from 65.9% (2006) to 72.8% (2016), *p* < 0.01 - Worse survival in patients receiving SLNB in 2012–2016 vs. 2006–2011 (*p* = 0.01)	SLNB use has increased, but patients in the later cohort (2012–2016) had worse survival, possibly due to more advanced disease and reduced chemotherapy

ALND: Axillary Lymph Node Dissection; DFS: Disease-Free Survival; LNE: Lymph Nodes Examined; MBC: Male Breast Cancer; NCDB: National Cancer Database; OS: Overall Survival; SEER: Surveillance, Epidemiology, and End Results Database; SLNB: Sentinel Lymph Node Biopsy.
